# A Rare Case of Late-Onset Ertapenem-Induced Encephalopathy

**DOI:** 10.7759/cureus.43713

**Published:** 2023-08-18

**Authors:** Alexander J Teague, Sushan Gupta, Vishesh Paul

**Affiliations:** 1 Internal Medicine, Carle Foundation Hospital, Urbana, USA; 2 Pulmonology and Critical Care, Carle Foundation Hospital, Urbana, USA

**Keywords:** ertapenem side-effect, ertapenem induced encephelopathy, ertapenem, drug-induced encephalopathy, drug-reaction

## Abstract

Encephalopathy is a rare side effect associated with carbapenem antibiotics, typically presenting within one week of initiating treatment. It is almost exclusively seen in patients with poor renal function. We present a case of a middle-aged male with a history of cerebral vascular accident and normal renal function admitted for agitation, delirium, and insomnia more than two weeks after starting ertapenem to treat osteomyelitis. He was empirically treated for meningitis on admission, and ertapenem was discontinued. After an extensive negative workup for infectious and neurological etiologies of encephalopathy, a presumptive diagnosis of ertapenem-induced encephalopathy was made. The patient returned to his baseline mental status five days after discontinuing ertapenem. The nature of his neurological symptoms and timely resolution after stopping ertapenem is consistent with ertapenem-induced encephalopathy and represents a notably delayed symptom onset compared to previously described cases.

## Introduction

Ertapenem is a commonly used broad-spectrum carbapenem antibiotic that is rarely associated with encephalopathy [1-4]. Seizures are the most common manifestation, but patients may also present with agitation, delirium, and insomnia symptoms, usually within one week of initiating therapy [2,3,5]. Risk factors for carbapenem-induced encephalopathy include renal impairment, low serum albumin, and prior central nervous system (CNS) injury [6-8]. Ertapenem-induced encephalopathy is often reversible, typically within 48 hours of drug cessation; however, recovery may be delayed up to two weeks in patients with severe renal dysfunction [2,5,9,10]. We present a case of delayed-onset encephalopathy manifesting in the third week after beginning ertapenem therapy for osteomyelitis in a patient with normal renal function.

## Case presentation

Our patient was a 51-year-old male who presented to the hospital with worsening confusion, hallucinations, and aggressive behavior. His medical history was significant for peripheral artery disease, uncontrolled type 2 diabetes mellitus (recent hemoglobin A1c of 9.7%), hypertension, and hyperlipidemia. His daily home medications included aspirin 81 mg daily, clopidogrel 75 mg daily, and metformin 1000 mg twice daily. His social history included active smoking with a 17 pack-year history and occasional alcohol consumption of about one beer per week. The rest personal and family history was non-contributory.

He had been recently hospitalized for a right foot diabetic ulcer and associated osteomyelitis, requiring a partial fifth-ray amputation with angioplasty and stenting of the superficial femoral artery. During that hospitalization, an infectious disease physician was consulted and recommended ertapenem after reviewing relevant tissue cultures and sensitivities. The patient was ultimately started on ertapenem and discharged with a plan to complete a four-week course. On day 17 of therapy, the patient developed worsening confusion and hallucinations. The primary care physician held the oxycodone and tramadol, suspecting opioid-related side effects. However, the patient's symptoms continued to worsen, and his family brought him to the emergency department for evaluation.

At the time of presentation, his vitals were: temperature-98.7 F, heart rate-130/minute, blood pressure-154/95 mmHg, and oxygen saturation-95% in room air. On exam day, he was restless and agitated and intermittently followed commands. The neurologic exam showed equal and reactive pupils bilaterally, symmetric facial expression, equal strength in all extremities, symmetric knee and ankle reflexes, and down-going Babinski bilaterally. The neck was supple without meningeal signs. The surgical incision site for the recent amputation did not show edema, erythema, discharge, or other signs of infection. The rest of the physical exam was unremarkable.

Initial laboratory studies revealed a mild leukocytosis of 11.98 10^3^/uL, normocytic anemia at 9.0 g/dL, and a normal platelet count. A comprehensive metabolic panel was notable for normal creatinine (0.87 mg/dL) and low serum albumin (2.9 g/dL). The complete blood count and comprehensive metabolic panel results are shown in Table 1. Other lab tests, including procalcitonin level, urinalysis, urine drug screen, ammonia level, and thyroid function, were also normal. A computed tomography (CT) scan of the brain showed evidence of an old small left frontal lobe infarction but no acute pathology. CT scan of the foot did not show any abscesses or gas formation. CT scan of the abdomen and pelvis ruled out any hidden focus of infection. 

**Table 1:** Laboratory results and reference ranges for the patient's complete blood count and comprehensive metabolic panel drawn at hospital admission.

Test	Value	Reference range
White blood cell count	11.80	(4.00–11.00 10^3^/u​L)
Hemoglobin	9.0	(12.0–18.0 g/dL)
Platelet	348	(140–400 10^3^/uL)
Sodium	141	(136–145 mmol/L)
Potassium	4.0	(3.5–5.1 mmol/L)
Chloride	106	(98–107 mmol/L)
Bicarbonate	20.0	(22.0–29.0 mmol/L)
Blood urea nitrogen	15	(8–26 mg/dL)
Creatinine	0.87	(0.55–1.30 mg/dL)
Calcium	8.9	(8.9–10.6 mg/dL)
Total protein	7.1	(6.0–8.0 g/dL)
Albumin	2.9	(3.5–5.0 g/dL)
Alkaline phosphatase	88	(40–150 U/L)
Aspartate aminotransferase	18	(5–34 U/L)
Alanine aminotransferase	17	(0–55 U/L)
Total bilirubin	1.0	(0.2–1.2 mg/dL)

With acute encephalopathy due to an unclear cause, a lumbar puncture was performed, and blood cultures were sent before the patient was started on empiric cefepime, ampicillin, vancomycin, and acyclovir. The ertapenem was stopped at this time. Cerebrospinal fluid (CSF) analysis showed a white blood cell count of 1/uL, a glucose level of 119 mg/dL, and a protein level of 32.3 mg/dL. A polymerase chain reaction panel covering 14 common bacterial, viral, and fungal causes of meningitis was negative. 

**Figure 1:**
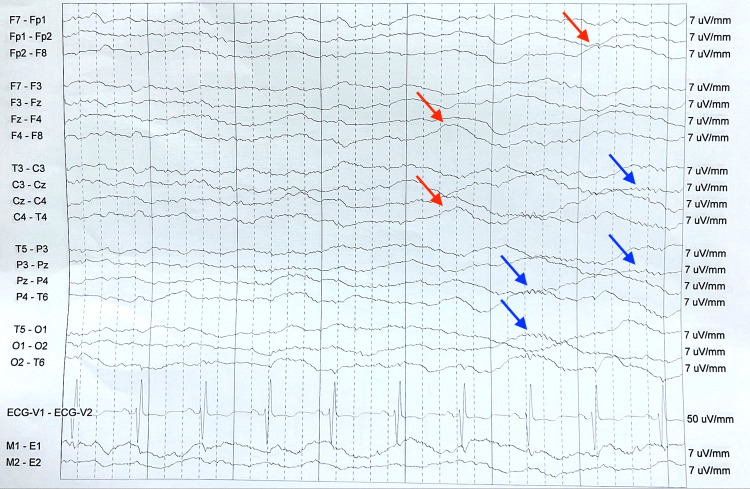
Representative segment of electroencephalogram recording. The above seven-second epochs were collected using a standard 10-20 electrode arrangement of continuous electroencephalogram (EEG) with accompanying video monitoring. The patient was under continuous propofol sedation at the time of the EEG. The EEG depicts brief one-second bursts of alpha and beta frequencies (blue arrows) on a background of diffuse suppression with low-amplitude delta slowing (red arrows), consistent with severe global encephalopathy or sedation.

Due to worsening agitation, the patient was transferred to the intensive care unit (ICU) and started on a dexmedetomidine drip, which worked well to control his agitation. Continuous electroencephalography (EEG) monitoring ruled out seizures and showed a global encephalopathy pattern (Figure 1). 

The next day, a magnetic resonance imaging (MRI) scan did not show signs of meningeal enhancement or any other acute process. The blood cultures and CSF cultures remained negative, and the empiric meningitis treatment was discontinued after 48 hours. The infectious disease team switched antibiotics to piperacillin-tazobactam to complete the treatment course for osteomyelitis. 

After excluding numerous possible infectious etiologies and acute neurologic pathologies, a presumptive diagnosis of ertapenem-induced encephalopathy was made. Supportive care was continued along with piperacillin-tazobactam for osteomyelitis and dexmedetomidine for agitation. The patient's encephalopathy started to clear by day four, and dexmedetomidine was successfully discontinued. By day five, he was back to normal mental status and was transferred out of the ICU. Eventually, he was discharged one week later without any complications and followed up with his primary care team. 

## Discussion

Encephalopathy with ertapenem use is rare and typically presents soon after initiating therapy. Ertapenem and its beta-lactam open-ring metabolite are antagonists to gamma-aminobutyric acid (GABA) type A receptors in the central nervous system, leading to CNS hyperarousal and associated side effects [2,9]. Seizures are the most common neurological side effect of ertapenem, but patients may also present with agitation, delirium, and insomnia, as in our case [2,3,5].

The drug has predominant renal excretion, so poor renal function is usually seen in patients with ertapenem-related side effects [3,11]. Most case reports describe symptoms beginning within one week of ertapenem administration [2-4,10]. Rarely, prolonged therapy may lead to drug accumulation despite normal renal function, resulting in delayed symptom onset, as in this case [10]. Additionally, low serum albumin at the time ertapenem is started has been associated with developing encephalopathy [8]. As ertapenem is highly protein-bound, low levels of albumin may lead to higher serum concentrations of free ertapenem, thus increasing the risk for adverse reactions [11]. Other patient-related factors associated with increased risk for ertapenem-associated seizures include old stroke (OR, 14.36; 95% CI, 4.38-47.02; p < 0.0001), brain imaging within one year of admission (OR, 5.73; 95% CI, 1.78-18.43; p = 0.0034), low hemoglobin level (OR, 3.88; 95% CI, 1.28-12.75; p = 0.0165), and thrombocytopenia (OR, 4.94; 95% CI, 1.56-15.68; p = 0.0067) [6]. Though this patient did not present with seizures, both seizures and encephalopathy represent CNS hyperarousal and thus may share similar risk factors. This patient's risk factors included low serum albumin, low hemoglobin, and evidence of a prior stroke.

The diagnosis of ertapenem-induced encephalopathy is challenging and often clinical. We had an extensive list of differential diagnoses in this patient with acute encephalopathy of unclear etiology. Altered sensorium due to narcotics was ruled out after his mental status didn't improve despite discontinuing the opioids. CSF analysis ruled out meningitis. EEG, CT scan, and brain MRI ruled out seizures and acute CNS pathology. Extensive evaluation with blood and urine cultures and CT scans of the abdomen, pelvis, and foot ruled out any infection as the cause of encephalopathy. A negative urine drug screen test and a normal ammonia level ruled out toxic and metabolic causes. Collectively, the labs and imaging thoroughly evaluated multiple causes of encephalopathy, including infectious and non-infectious, all of which were unrevealing, thus strongly suggesting ertapenem encephalopathy. Ertapenem blood levels were not checked in the patient as they have not been shown to correlate with ertapenem's side effects.

The Naranjo Scale is a validated approach to estimating the probability of adverse reactions due to a drug [12]. Retrospectively evaluating our patient, the Naranjo Scale was seven, suggesting that ertapenem-induced encephalopathy was probable (Table 2).

**Table 2:** Naranjo adverse reaction probability scale. The Naranjo score for a suspected drug reaction is calculated by answering each of the 10 questions with a 'yes,' 'no,' or 'do not know' response. Each response has an associated score, which is summed to yield the total Naranjo score. The scores range from −4 to 12, with a higher score suggesting a more likely adverse drug reaction. The likelihood of an adverse drug reaction can be interpreted as doubtful (<0), possible (0 to 4), probable (5 to 8), and definite (>8) [12]. In this case, the Naranjo score for suspected ertapenem encephalopathy was seven, consistent with a probable drug reaction.

Questions	Yes	No	Do not know	Ertapenem encephalopathy
Are there previous conclusive reports about the reaction?	+1	0	0	+1
Did the adverse event appear after the suspected drug was administered?	+2	−1	0	+2
Did the adverse reaction improve after the drug was discontinued or after a specific antagonist was administered?	+1	0	0	+1
Did the adverse event reappear when the drug was re-administered?	+2	−1	0	0
Are there alternative causes that could on their own have caused the reaction?	−1	+2	0	+2
Did the reaction reappear when a placebo was given?	−1	+1	0	0
Was the drug detected in blood (or other fluids) in concentrations known to be toxic?	+1	0	0	0 (Unknown)
Was the reaction more severe when the dose was increased or less severe with the dose was decreased?	+1	0	0	0 (Unknown)
Did the patient have a similar reaction to the same or similar drugs in any previous exposure?	+1	0	0	0 (Unknown)
What is the adverse event confirmed by any objective evidence?	+1	0	0	+1
Total score:				7

Symptoms of ertapenem-induced encephalopathy typically improve within two to 14 days after stopping therapy [2]. Our patient recovered to his baseline mental status five days after stopping treatment. The prompt recovery after discontinuing ertapenem and an extensive negative infectious and non-infectious evaluation strongly support our patient's diagnosis of ertapenem-induced encephalopathy. 

The patient ultimately required minimal medical intervention and recovered with no known sequela. The patient was interviewed after he was at baseline mental status, and his perspective of the sequence of events is mentioned in the Appendix. 

## Conclusions

Encephalopathy is a rare side effect of ertapenem and can present within the first few days to weeks of starting treatment. Due to its similar presentation, meningitis is usually the top differential and needs to be ruled out. Medical providers should remain cognizant of this side effect in a patient on ertapenem therapy after excluding other infectious and non-infectious etiologies. In particular, our case illustrates that ertapenem-induced encephalopathy may present anytime during therapy, even in patients with normal renal function. Improved awareness of ertapenem-associated encephalopathy may result in earlier detection, intervention, and resolution.

## Appendices

Patient perspective

After the patient returned to his baseline mental status, he was asked to describe his entire experience with ertapenem encephalopathy:

"As I know, when I got home from the hospital (after my foot infection), I sat at home and all of a sudden, I started getting these seizures. It's kind of hard to explain. I started getting mad and I started getting really upset with my wife. I was threatening my wife and everything else. Then my wife took me to the hospital, and she told me that there was something wrong with me and I needed to get it fixed. So, they put me in the hospital, and I sat in the hospital for the longest time. Then all of a sudden, to tell you the truth, I died and I didn't wake up until the beginning of (the month). I kind of wasted one-quarter of my (month). Just to think about this really upsets me. If there is anything I can do to help this situation, it is going to help (me). Thanks."

Of note, there was no documentation of seizures in the patient's medical record. Even when history was obtained from the patient's wife at multiple separate points during his presentation, there was no mention of seizure activity from her. It is unclear exactly what the patient meant by this statement.
